# Comparative safety and efficacy of new-generation single-layer polytetrafluorethylene- versus polyurethane-covered stents in patients with coronary artery perforation for the RECOVER (REsults after percutaneous interventions with COVERed stents) Investigators

**DOI:** 10.1007/s12928-025-01084-y

**Published:** 2025-02-07

**Authors:** Felix Voll, Göran Olivecronab, Miroslaw Ferenc, Farrel Hellig, Christian Schlundt, Jochen Wöhrle, Salvatore Cassese, Wolfgang Rottbauer, Adam Witkowski, Erion Xhepa, Wiktor Kuliczkowski, Lisa Strauss, Benedikt Schrage, Michael Joner, Constantin von zur Mühlen, Stephane Cook, Tomislav Miljak, Holger Eggebrecht, Eric Eeckhout, Karl-Ludwig Laugwitz, Jacques Monsegu, Heribert Schunkert, Dirk Westermann, Adnan Kastrati, Nicolas Dumonteil, Ralf Birkemeyer, Sebastian Kufner

**Affiliations:** 1 Deutsches Herzzentrum München, TUM Universitätsklinikum, Munich, Germany; 2 University of Erlangen, Erlangen, Germany; 3 Klinikum Rechts Der Isar, TUM Universitätsklinikum, Munich, Germany; 4 Herzklinik Ulm, Ulm, Germany; 5 Lund University, Skane University Hospital, Lund, Sweden; 6 Sunninghill Hospital, Johannesburg, South Africa; 7 Ulm University Heart Center, Ulm, Germany; 8 Medical Campus Lake Constance, Friedrichshafen, Germany; 9 Institute of Cardiology, Warsaw, Poland; 10 Institute for Heart Diseases, Wroclaw Medical University, Wroclaw, Poland; 11 University Heart Center Hamburg, Hamburg, Germany; 12 Hospital Fribourg, Fribourg, Switzerland; 13 Klinikverbund Südwest: Zentrum Für Kardiologie Nagold, Herrenberg, Germany; 14 Cardioangiologisches Centrum Bethanien, Frankfurt, Germany; 15 Centre Hospitalier Universitaire Vaudoise, Lausanne, Switzerland; 16 Groupe Hospitalier Mutualiste de Grenoble, Grenoble, France; 17 Clinique Pasteur, Toulouse, France; 18 DZHK (German Centre for Cardiovascular Research), Partner Site Munich Heart Alliance, Munich, Germany; 19 Present Address: Universitäts-Herzzentrum Freiburg . Bad Krozingen, Freiburg, Germany

**Keywords:** Coronary artery perforation, Covered stent, Polytetraflourethylene, Polyurethane

## Abstract

New-generation single-layer polytetrafluorethylene (PTFE-) or polyurethane (PU-) covered stent (CS) for the treatment of coronary artery perforation (CAP) during PCI offer high procedural efficacy. To evaluate the comparative long-term safety and efficacy of both devices. This is a multicenter pooled analysis of individual data of patients with CAP undergoing implantation of single-layer PTFE-CS or PU-CS. Procedural endpoint was strategy success defined as successful placement of CS and sealing of perforation without surgical conversion. Clinical endpoints were mortality, myocardial infarction (MI), target vessel revascularization (TVR) and definite or probable stent thrombosis (def/prob ST) at 12 months. Seventy patients with CAP underwent implantation of two hundred eight CS, ninety-two PTFE-CS, and one hundred sixteen PU-CS. More than 1 stent was implanted in 13 patients (17.1%) in PTFE-CS group and 19 patients (20.2%) in PU-CS group, *P* = 0.80. Strategy success was high (96.1% versus 92.5%., *P* = 0.62). At 12 months, 71 patients (93.2%) in PTFE-CS group versus 79 patients (81%) in the PU-CS were alive, *P* = 0.05; TVR occurred in 14 patients (28.4%) in PTFE-CS group and 12 patients (17.9%) in PU-CS group, *P*= 0.54; MI in 1 patient (1.3%) in PTFE-CS group and 1 patients (1.1%) in PU-CS group, *P* = 0.86. Rates of def/prob ST were comparable 1.3% in PTFE-CS versus 3.1% in PU-CS *P* = 0.95. A strategy of implantation of a new-generation single-layer PTFE- or PU-CS for the treatment of coronary artery perforation showed high success rates. Both new-generation CS showed favorable and similar clinical safety, in particular with regard to thrombotic events.

**Graphical abstract:**

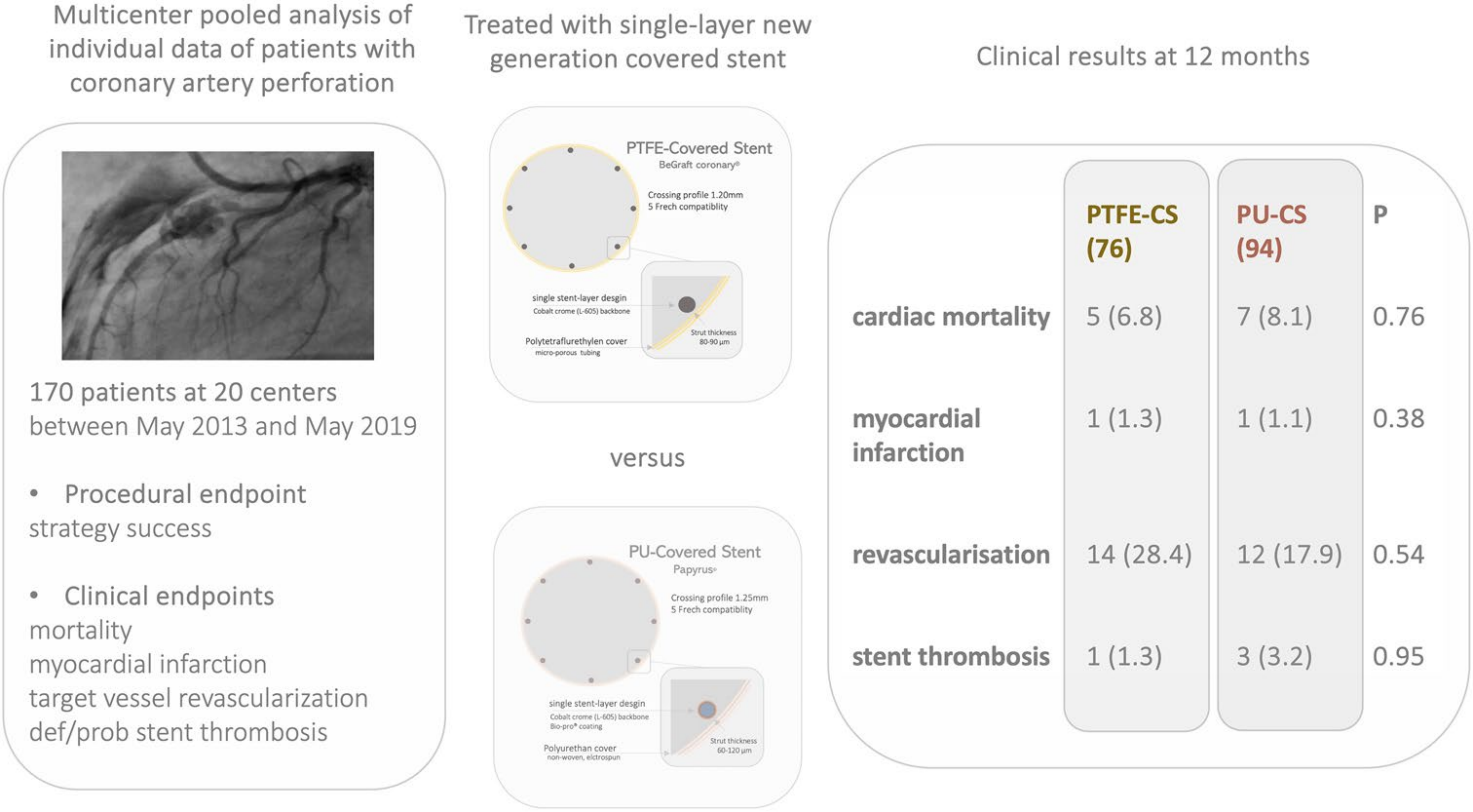

**Supplementary Information:**

The online version contains supplementary material available at 10.1007/s12928-025-01084-y.

## Introduction

Covered stent (CS) have become a cornerstone in the treatment of coronary artery perforation (CAP), a feared complication during percutaneous coronary intervention (PCI) [1]. Although, notwithstanding the implantation of CS has proven to be potentially life-saving, concerns exist with regard to increased rates of thrombotic events and limited long-term anti-restenotic efficacy of CS.

In particular, early generation CS with thicker crossing profile and PTFE coverage captured between two stent-strut layers have been associated with increased risk of periprocedural and postprocedural thrombotic events. Recently, rates of stent-thrombosis have been reported to be threefold higher in patients treated with early double PTFE layer CS as compared to new-generation single-layer polyurethane-CS [2].

In addition, mid- to long-term efficacy of double-layer PTFE-CS was associated with increased revascularization rates, with reported device failure in up to every third patient [3–5].

Based on fundamental iterations in stent design, current available new-generation single-layer polyurethane or PTFE-CS seem to have overcome some of these major drawbacks of their early generation counterparts [2]. Recently published data of patients treated with FDA approved new-generation single-layer polyurethane-CS suggest favorable procedural and clinical efficacy and safety profile [6]. Previously, we reported favorable, case-series-based data, concerning angiographic efficacy and clinical event rates after treatment with new-generation single-layer PTFE-CS [7–9]. Nevertheless current data could not fully rule out concerns about thrombotic complications especially concerning single-layer polyurethane-CS with reported ST rates as high as 10% [10]. In this vein, the comparative efficacy and safety of new-generation single-layer PTFE- versus polyurethane-CS are of particular interest.

Therefore, we performed the current pooled analysis based on individual patient data from three dedicated registries to investigate procedural and clinical outcomes after treatment of coronary perforations with new-generation single-layer PTFE-CS versus polyurethane-CS [7, 8]. [11]

## Methods

### Population

In this retrospective pooled analysis, we included individual patient data of 170 patients undergoing implantation of 208 CS (single-layer PTFE-CS (BeGraft coronary stent, Bentley InnoMed GmbH, Hechingen, Germany or PU-CS (PK Papyrus, Biotronik AG, Buelach, Switzerland)) in native coronary arteries or aortocoronary bypass graft, in 20 centers, between May 2013 and May 2019 [7, 8, 11]. Enrolling centers are summarized in supplemental Table 1.

**Table 1:** Baseline demographic and clinical characteristics

Patient characteristics (*n*)	PTFE -CS (*n* = 76)	PU-CS (*n* = 94)	Overall (*n* = 170)	*P* value
Sex				> 0.99
Male, n (%)	54 (45)	66 (55)	120 (71)	
Female, n (%)	22 (44.9)	27 (55.1)	49 (28.9)	
Age (y)	71.6 (69.4–73.8)	72.8 (70.5–75.1)	72.4 (70.4–74.9)	0.463
Diabetes mellitus, n (%)	19 (25)	23 (24.5)	42 (24.7)	> 0.99
Insulin, n (%)	4 (5.7)	10 (10.6)	14 (8.5)	0.398
art. Hypertension	65 (85.5)	62 (65.9)	127 (74.7)	**0.004**
Hypercholesterinemia, n (%)	49 (52.1)	n.a		
Current smoker, n (%)	13 (17.1)	17 (18.1)	30 (17.6)	> 0.99
Previous MI, n (%)	17 (22.4)	33 (35.1)	50 (29.4)	0.09
Previous PCI, n (%)	41 (52.5)	37 (39.3)	78 (45.8)	0.065
Previous CABG, n (%)	15 (19.7)	19 (20.2)	34 (20)	> 0.99
Clinical presentation *n* (%)				
Stable angina	60 (68.9)	27 (28.7)	87 (51.1)	** < 0.001**
NSTEMI	11 (15.9)	28 (29.8)	39 (23.9)	**0.043**
STEMI	3 (4.3)	17 (18.0)	20 (12.2)	**0.008**

Data are shown as absolute numbers and percentage (%) or mean ± standard deviation or interquartile range 25–75%. PCI = percutaneous coronary intervention; *CABG*   coronary artery bypass graft, *PTFE* polytetrafluoroethylene, *PU*   polyurethane, *CS*   covered stent p<0.05 (in bold)

### Study devices

The BeGraft coronary stent (Bentley InnoMed GmbH, Hechingen, Germany) is a Cobalt Chrome (L-605), open-cell platform covered with a single layer of a microporous PTFE membrane (thickness of 89 ± 25 μm). The membrane is fixed at the proximal and distal stent ends, providing a 100% coverage of the stent surface area. The flexible single-layer design in combination with a low-profile balloon catheter result in viable crossing profile between 1.1—1.4 mm with a guiding catheter compatibility of 5 French for all sizes.

**Fig. 1:**
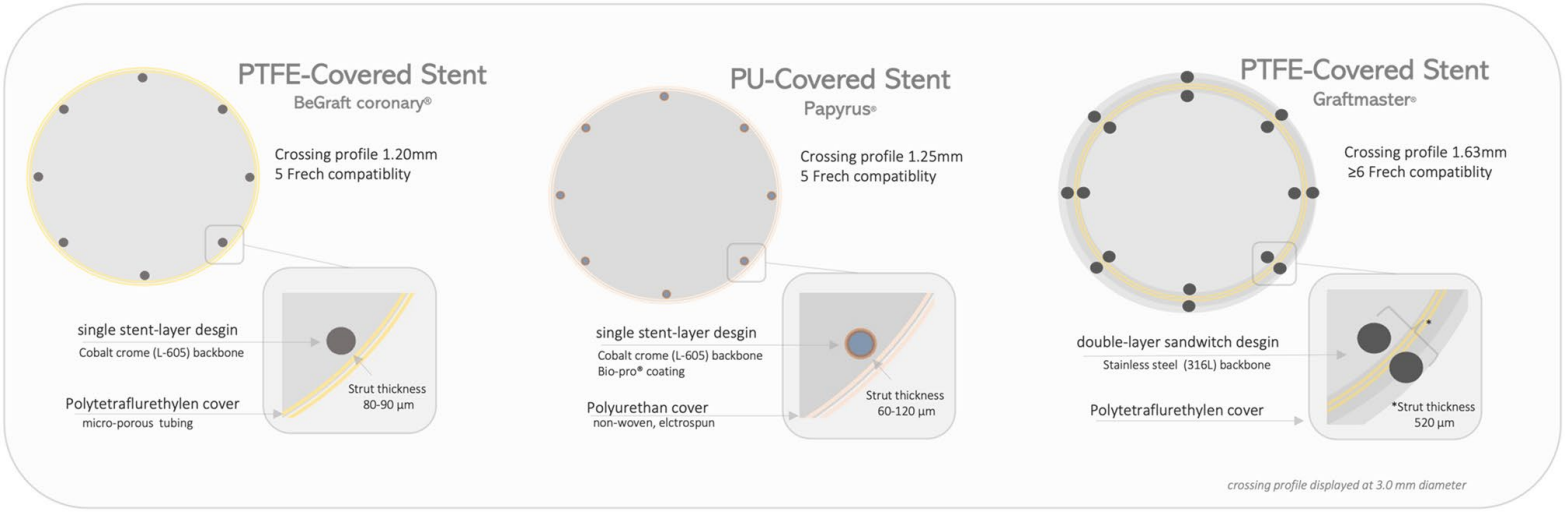
New-generation single-layer covered stents with polyurethane or PTFE cover compared to early generation sandwich design PTFE-covered stent

The PK Papyrus-covered stent system (Biotronik AG, Buelach, Switzerland) consists of covered single-layer stent. The stent body is made of cobalt-chromium and is the same as that used for the Orsiro drug-eluting stent (Biotronik AG), with ultrathin struts of 60 μm (≥ 3.5 mm Ø 80 μm). The electrospun cover consists of non-woven, small fibers made of polyurethane, a biostable polymer. The crossing profile of PK Papyrus is 1.25 mm for Ø 3.0 mm, and the delivery system is 5F compatible (≥ Ø 4.5 mm 6F). A detailed description of both new-generation single-layer devices as compared to early generation sandwich design device is displayed in Fig. 1.

### Statistical analysis

Continuous data are presented as mean (SD). Categorical data are presented as counts or proportions (%). Differences between groups were checked for significance using Student’s *t* test for continuous data and Chi-squared test (or Fisher’s exact test where the expected cell value was < 5) for categorical variables. In significant differences in the baseline lesion and patient characteristics, we performed an adjusted analysis. Adjusted analysis was performed accounting for the following variables: clinical presentation NSTE-ACS (yes/no), chronic total occlusion (CTO, yes/no), calcification grade (> moderate, yes/no). Statistical software STATA Version 11.2, StataCorp LP Texas, USA was used for all other analyses.

### Data management, endpoints, and definitions

Ellis classification was used for definition and classification of coronary artery perforation [12]. Type I coronary artery perforation is defined by the development of an extraluminal crater without extravasation. Type II coronary artery perforation is defined by the development of a pericardial or myocardial blush without contrast jet extravasation. Type III coronary artery perforation is defined by the development of an extravasation jet through a frank (> 1 mm) perforation or cavity spilling into an anatomic cavity chamber. Cardiac tamponade is defined as pericardial effusion proofed by echocardiography, in addition to clinical signs of acute tamponade, including hypotension, tachycardia, and cardiogenic shock.

Procedural endpoint of interest is strategy success defined as successful placement of covered stent and sealing of perforation (defined as the angiographic absence of any contrast medium extravasation due to vessel perforation or rupture after implantation of covered stent) without acute deterioration of the clinical status of the patient and/or need for urgent surgical intervention.

CAC was graded into none, mild, moderate, and severe according to the angiographic classification of Mintz et al [13].

Clinical endpoints of interest were all-cause mortality, spontaneous myocardial infarction (MI), and target vessel revascularization (TVR) at 12 months. Myocardial infarction (MI) was defined according to the fourth universal definition of MI [14]; TVR was defined as any angina-driven repeated percutaneous coronary intervention of the target vessel. Other endpoints of interest were cardiac mortality, target lesion revascularization (TLR) defined as any angina driven repeated percutaneous coronary intervention of the target lesion (the previously treated segment + 5 mm), periprocedural MI, defined as any CK-MB (or CK) elevation ≥ 3times of the upper limit of normal (ULN) and elevation of at least 50% over the most recent pre-PCI levels or new ECG changes consistent with MI within the first 24 h after the procedure and definite or probable stent thrombosis (def/prob ST) at 12 months according to the definition of the academic research consortium (ARC) [15]. Anonymized angiographic films and source data, as far as available, were analyzed by two interventional cardiologists. In the case of divergent evaluations, a consensus meeting was arranged.

## Results

### Patient baseline and demographic characteristics

In this analysis, we included individual patient data of 170 patients undergoing implantation of 208 single-layer CS. Seventy-six patients underwent implantation of ninety-two single-layer PTFE-CS and ninety-four patients underwent implantation of one hundred sixteen PU-CS in native coronary arteries or aortocoronary bypass grafts after perforation.

Baseline characteristics were well balanced between the two groups. Despite, patients treated with PTFE-covered stent had more often arterial hypertension and presented less often with an acute coronary syndrome as compared to patients treated with PU-covered stent (hypertension PTFE-CS: 85.5% versus PU-CS 85.5%, *P* = 0.004; NSTEMI: PTFE-CS: 15.9% versus PU-CS 29.8%, *P* = 0.043; STEMI: PTFE-CS: 4.3% versus PU-CS 18.0%, *P* = 0.008). Patient baseline and demographic characteristics according to treatment group are summarized in Table 1.

### Baseline lesion and procedural characteristics

Baseline lesion and procedural characteristics were well balanced between the two groups. Most patients had complex lesions characteristics with moderate-severe calcification in over 80% of cases. Significantly more perforations in the PTFE-CS group as compared with PU-CS group occurred in the context of chronic total occlusion lesions (51.3% in the PTFE-CS group versus 8.5% in the PU-CS group, *P* < 0.001). The majority of perforation lesions were located in native coronary arteries (92.7%), nine lesions were located in saphenous vein graft (5.3%), two lesions (1.1%) in arterial coronary bypass graft. The vast majority of patients treated with PTFE-CS experienced Ellis grade III coronary perforations (85.5%) as compared to lower rates in PU-CS group (72.3%) resulting in a significant difference in Ellis grade distribution between the two groups, *P* = 0.042. Rates of cardiac tamponade, however, were similar between the two groups (27.6% in both groups, *P* > 0.99). An urgent pericardiocentesis to treat or to prevent cardiac tamponade was performed in 30.2% of PTFE-CS cases and 24.4% of PU-CS cases, *P* = 0.488.

Concerning the mechanism of vascular injury, resulting in coronary perforation, the main cause of perforation was balloon pre-dilation or balloon dilation during drug-eluting stent implantation in all patients (overall 84.6%). However, guidewire-induced injuries were more frequent in PTFE-CS group (18.4%) as compared to PU-CS group (7.4%), *P* = 0.036. Balloon dilation-induced perforations were significantly less frequent in PTFE-CS group (27.6%) as compared to PU-CS group (47.8%), *P* = 0.08 and rates of stent-induced perforations were comparable between PTFE-CS group (51.3%) versus PU-CS group (41.4%), *P* = 0.218. Lesion and procedural characteristics according to treatment group are summarized in Table 2.

**Table 2:** Lesion and procedural characteristics

Lesion characteristics	PTFE -CS (n = 76)	PU-CS (n = 94)	All (170)	*P* value
*Vessel treated (%)*				0.447
LAD	39 (51.3)	44 (46.8)	83 (48.8)	
LCx	14 (45.1)	17 (54.8)	31 (18.2)	
RCA	21 (27.6)	24 (25.5)	45 (26.4)	
ACVB	2 (2.6)	7 (7.4)	9 (5.3)	
art. graft	0 (0.0)	2 (2.1)	2 (1.1)	
*Calcification, n (%)*				
None	9 (11.8)	9 (9.5)	18 (10.6)	0.803
Mild	13 (17.1)	0 (0.0)	13 (7.6)	** < 0.001**
moderate	26 (34.2)	47 (50)	73 (42.9)	**0.044**
severe	28 (36.8)	38 (40.)	66 (38.8)	0.752
CTO, n (%)	39 (51.3)	8 (8.51)	47 (27.6)	** < 0.001**
Pericardial effusion, n (%)	49 (65.3)	n.a		
Pericardial tamponade, n (%)	21 (27.6)	26 (27.6)	47 (27.6)	> 0.99
Pericardiocentesis, n (%)	23 (30.2)	23 (24.4)	46 (27)	0.488
*Ellis-Classification, n (%)*				
Ellis grade I	2 (2.1)	2 (2.6)	4 (2.4)	> 0.99
Ellis grade II	8 (10.5)	24 (25.5)	32 (18.8)	**0.017**
Ellis grade III	65 (85.5)	68 (72.3)	133 (78.2)	**0.042**
Mechanism of vascular injury, n (%)				
Guidewire	14 (18.4)	7 (7.4)	21 (12.3)	**0.036**
Balloon	21 (27.6)	45 (47.8)	66 (38.8)	**0.008**
Stent	39 (51.3)	39 (41.4)	78 (45.8)	0.218
Other	2 (2.1)	8 (8.5)	10 (5.9)	0.063

Data are shown as absolute numbers and percentage (%), *ACVB*   aortocoronary venous bypass graft, *CTO* chronic total occlusion, *PTFE*  polytetrafluoroethylene, *PU*   polyurethane, *CS*   covered stent p<0.05 (in bold)

### Covered stent implantation procedural characteristics

The number of CS implanted by lesion was 1.2. More than 1 CS was implanted in 32 cases (18.8%), 13 cases (17.1%) in PTFE-CS group and 19 cases (20.2%), *P* = 0.80. The maximum number of CS implanted by case was three in the PTFE-CS group and four in the PU-CS group. Both in cases of geographical miss. Total stented length was significant higher in PTFE-CS group as compared to PU-CS group 43.9 ± 32mm versus 24.4 ± 11.1mm, *P* < 0.001. Detailed results are displayed in Table 3.

**Table 3:** Covered stent implantation procedural characteristics

	PTFE -CS (*n* = 76)	PU-CS (*n* = 94)	All (170)	*P* value
Procedural characteristics				
Number of CS	1.2 (	1.2	1.2	0.997
max. implantation press. (atm)*	16.0 (± 4.9)	14.5 (± 3.3)	15.2 (± 4.2)	**0.019**
max. stent diameter** (mm)	3.2 (± 0.6)	3.2 (± 0.6)	3.2 (± 0.6)	0.6
Total stent length (mm)	43.9 (± 32)	24.4 (± 11.1)	33.1 (± 24.8)	** < 0.001**
Procedural success				
Strategy success	73 (96.1)	87 (92.5)	160 (94.1)	0.623
Successful placement of CS	76 (100)	94 (100)	170 (100)	> 0.99
Sealing success	73 (96.1)	88 (93.6)	161 (94.7)	0.733
Surgical conversion	0	7 (7.4)	7 (4.1)	

Data shown as number (…), mean ± SD or number (percentage) based on in-segment analysis, data available in *(PTFE-CS: 72/76; PU-CS: 85/94); ** (PTFE-CS: 75/76; PU-CS: 88/94) p<0.05 (in bold)

### Procedural outcome

Strategic success was achieved in all but ten cases (5.9%) and comparable frequently achieved in patients treated with PTFE-CS (96.1%) and patients treated with PU-CS (92.6%), *P* = 0.323. Rates of successful placement of covered stent and sealing of perforation (defined as the angiographic absence of any contrast medium extravasation due to vessel perforation or rupture after implantation of covered stent) were comparable in patients treated with PTFE-CS versus PU-CS (96.0% versus 93.6%, *P* = 0.733).

Deterioration of the clinical status of any patient with the need for urgent surgical intervention was observed in three cases with unsuccessful and four cases with successful sealing of the coronary perforation, all in PU-CS group. Detailed results are displayed in Table 3.

### Clinical outcome

**Fig. 2:**
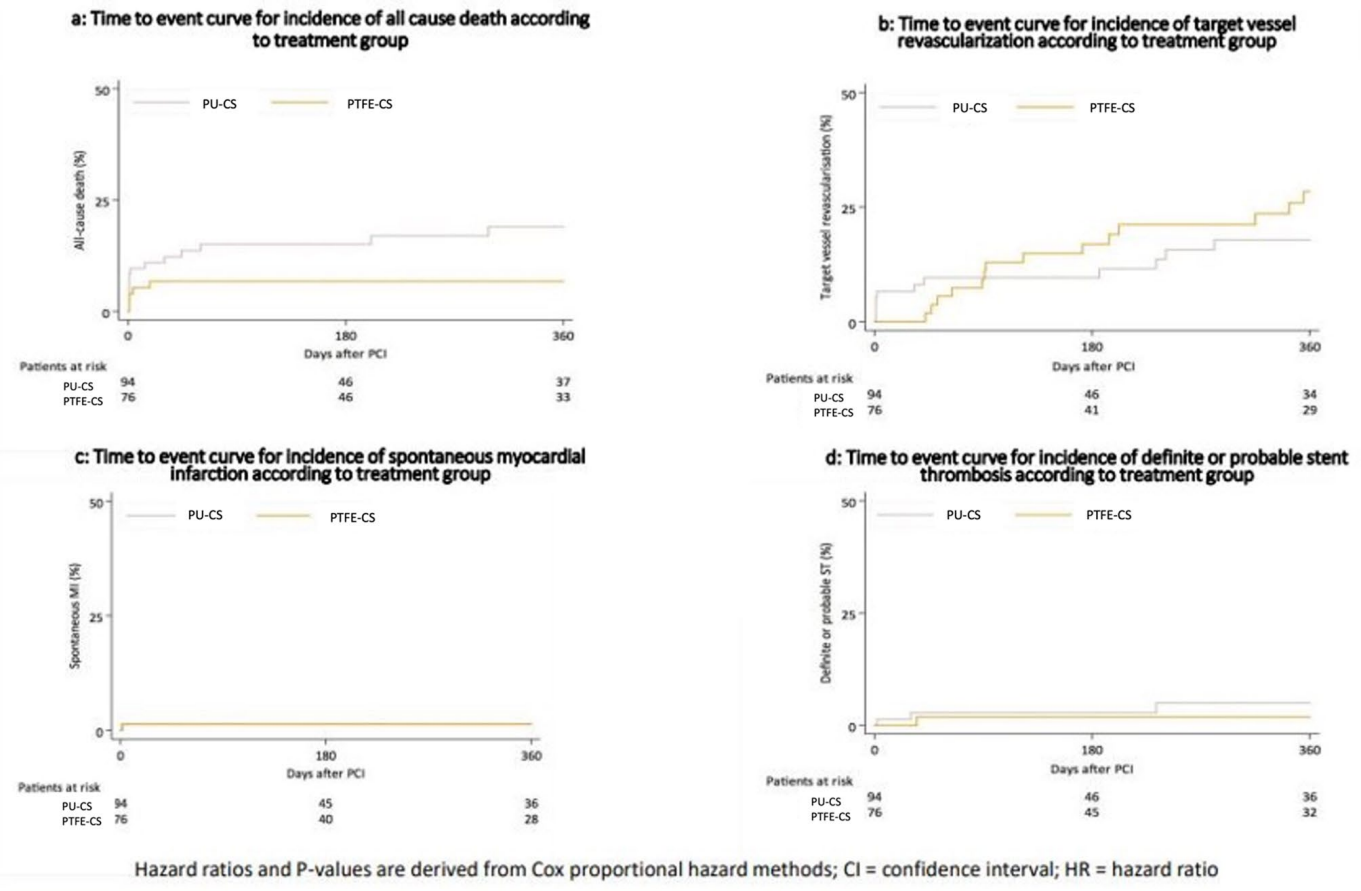
**a** Time to event curve for incidence of all-cause death according to treatment group. **b** Time to event curve for incidence of target vessel revascularization according to treatment group. **c** Time to event curve for incidence of spontaneous myocardial infarction according to treatment group. **d** Time to event curve for incidence of definite or probable stent thrombosis according to treatment group. a-d: Hazard ratios and *P* values are derived from Cox proportional hazard methods; *CI* confidence interval, *HR* hazard ratio

Clinical event rates were high but comparable between the groups. Clinical outcome is displayed in detail in Table 4. Results for the adjusted analysis (after adjustment for the factors clinical presentation, chronic total occlusion and calcification) are displayed in Table 5. At 12 months, 71 patients in PTFE-CS group versus 79 patients in the PU-CS were alive, (93.2%% versus 81% respectively, hazard ratio (HR) = 0.39, 95% confidence interval (CI) 0.1–1.1; *P* = 0.068; hazard ratio _adjusted_ (HR_adj._) = 0.67, 95% confidence interval (CI) 0.2–2.0;* P* = 0.472). Time to event curve is displayed in Fig. 2.

**Table 4:** Clinical outcome according to treatment group

	PTFE-CS (76)	PU-CS (94)	All (170)	HR 95% CI	*P* value
Mortality					
All-cause death	5 (6.8)	15 (19.0)	20 (11.8)	0.39 (0.1–1.1)	0.068
Cardiac death	5 (6.8)	7 (8.1)	12 (7.0)	0.84 (0.3–2.6)	0.763
Myocardial infarction (MI)					
Spontaneous MI	1 (1.3)	1 (1.1)	3 (1.8)	1.28 (0.8–20.4)	0.864
Procedural MI	20 (26.3)	8 (8.51)	28 (16.4)	3.09 (1.4–7.0)	**0.007**
Revascularization					
TVR	14 (28.4)	12 (17.9)	26 (15.3)	1.33 (0.6–2.9)	0.471
TLR	11 (21.9)	6 (9.9)	17 (10.0)	2.10 (0.8–5.7)	0.147
Stent thrombosis					
Definite ST	1 (1.3)	2 (2.1)	3 (1.8)	0.56 (0.5–6.2)	0.635
Definite or probable ST	1 (1.3)	3 (3.2)	4 (2.4)	0.37 (0.4–3.6)	0.374

Data shown as number (percentage based on Kaplan––Maier estimates), hazard ratio (HR), confidence interval (CI) p<0.05 (in bold)

**Table 5:** Adjusted analysis, clinical outcome according to treatment group

	PTFE-CS (76)	PU-CS (94)	adj.HR 95% CI	*P* value
Mortality				
All-cause death	5 (6.8)	15 (19.0)	0.67 (0.2–2.0)	0.472
Cardiac death	5 (6.8)	7 (8.1)	0.84 (0.3–2.6)	0.763
Myocardial infarction (MI)				
Spontaneous MI	1 (1.3)	1 (1.1)	3.54 (0.2–60.1)	0.383
Procedural MI	20 (26.3)	8 (8.51)	3.94 (1.6–9.8)	0.003
Revascularization				
TVR	14 (28.4)	12 (17.9)	1.33 (0.5–3.3)	0.540
TLR	11 (21.9)	6 (9.9)	1.66 (0.5–5.3)	0.394
Stent thrombosis				
Definite ST	1 (1.3)	2 (2.1)	1.19 (0.1–13.1)	0.889
Definite or probable ST	1 (1.3)	3 (3.2)	0.93 (0.1–9.2)	0.951

Data shown as number (percentage based on Kaplan–Maier estimates), adjusted hazard ratio (adj.HR), Confidence interval (CI), adjusted for the factors clinical presentation, chronic total occlusion, calcification

The PTFE-CS in comparison with the PU-CS showed similar risk of cardiac death (6.8% versus 8.1% respectively, HR = 0.84, 95% CI 0.3–2.6;* P* = 0.763; HR_adj._ = 0.84, 95% CI 0.3–2.6;* P* = 0.763) and spontaneous MI (1.3% versus 1.1% respectively, HR = 1.28, 95% CI 0.79–20.4; *P* = 0.864; HR_adj._ = 3.54, 95% CI 0.2–60.1;* P* = 0.383). Time to event curve is displayed in Fig. 2.

Rates of periprocedural MI were higher in patients treated with PTFE-CS in comparison with the PU-CS (26.3% versus 8.51% respectively) in crude analysis (HR = 3.09, 95% CI 1.4–7.0; *P* = 0.007) and after adjustment (HR_adj._ = 3.94, 95% CI 1.6–9.8;* P* = 0.003).

Concerning revascularization, rates were numerically higher in patients treated with PTFE-CS as compared to PU-CS without statistically significant difference, in both TVR (28.4% versus 17.9% respectively, HR = 1.33, 95% CI 0.6–2.9; *P* = 0.471; HR_adj._ = 1.33, 95% CI 0.5–3.3;* P* = 0.540) and TLR (21.9% versus 9.9% respectively, HR = 2.1, 95% CI 0.8–5.7; *P* = 0.147; HR_adj._ = 1.66, 95% CI 0.5–5.3;* P* = 0.394). Time to event curve is displayed in Fig. 2.

Concerning thrombotic events, rates were low and comparable between the two groups, concerning both definite ST (1.3% versus 2.1% respectively, HR = 0.56, 95% CI 0.5–6.2; *P* = 0.635; HR_adj._ = 1.19, 95% CI 0.1–13.1;* P* = 0.889) and definite or probable ST (1.3% versus 3.2% respectively, HR = 0.37, 95% CI 0.4–3.6; *P* = 0.374; HR_adj._ = 0.93, 95% CI 0.1–9.2;* P* = 0.951). Time to event curve is displayed in Fig. 2.

## Discussion

This unique analysis is the first comparison of procedural and clinical efficacy and safety of new-generation single-layer covered stents with polytetrafluorethylene versus polyurethane cover in patients with coronary perforation during PCI.

The main findings of this dedicated retrospective analysis, based on individual patient data, can be summarized as follows:

First, in this study, both devices are associated with high and comparable procedural success rate. This finding is based on the high deliverability of both devices, the high rates of sealing success after CS implantation, and low rates of necessary surgical conversion.

Second, clinical event rates including mortality remain considerable and reflect the course of events after PCI complicated by coronary perforation.

Third, considering anti-restenotic efficacy, both polytetrafluorethylene- and polyurethane-covered stent showed comparable but considerable revascularization rates.

Fourth, the overall low and comparable rate of thrombotic events including myocardial infarction and stent thrombosis indicates a high safety profile of both new-generation single-layer CS irrespective of the cover material.

CS are potentially life-saving devices. However, once an emergency scenario is overcome, the device remains in the patient for a complete life span. Therefore, important considerations associated with the use of CS include an ongoing debate on their limited anti-restenotic efficacy. Studies report that stent restenosis in CS occurs with a frequency of 30–50% within the first 6 months. [16–19] A contributing factor might be the synthetic cover-membrane triggering excessive neointimal proliferation. While novel pericardium CS were not able to overcome current limitations with respect to anti-restenotic efficacy [20], polyurethane-CS report TLR rates as low as 4–6%. In line, PU-CS has gained FDA approval in 2018 and consequently increasingly replaced first-generation CS. We previously reported high anti-restenotic efficacy of new-generation single-layer PTFE-CS (revascularization rates 7.7%) [11], In line, concerning the angiographic efficacy of new-generation single-layer PTFE-CS, low late-lumen loss and acceptable rates of binary angiographic restenosis rates have been reported previously. [7, 11] In this analysis, target vessel revascularization rates range from 18–28% and were numerically higher in the PTFE-covered stent groups. Although these findings are broadly in line with results from previous trials [7, 10, 11], these findings deserve further considerations. First, given the emergent character of CS procedures, TVR rates may not only reflect device efficacy rather than the lack of optimal upfront lesion preparation and post-dilation once CS is implanted. Therefore, adequate post-dilation and procedural guidance with intravascular imaging could lead to a better apposition and expansion of the CS and consequently may improve clinical outcomes. Second, given the small number of patients in this analysis, from two cohorts with differences in lesion and procedural characteristics, conclusions concerning anti-restenotic efficacy should be drawn with caution. Nevertheless, after adjustment both devices, PU- and PTFE-CS, respectively, showed comparable anti-restenotic efficacy. This suggests that favorable outcomes concerning revascularization rates may drive predominantly from the single-layer design of new-generation CS rather than the membrane material.

Overall new-generation single-layer CS seem to have in part overcome previous drawbacks associated with early generation double-layer CS. This is specifically true, concerning rates of thrombotic events. Acute spontaneous myocardial infarction at 12 months occurred in 1.8% of patients with similar results concerning PU- versus PTFE-CS. On the other hand, the difference concerning procedural MI with significant higher event rates in the PTFE-CS group should be interpreted in detail. Both cohorts used comparable but not similar definitions of periprocedural MI. Although this analysis is based on the same definition of periprocedural MI and individual patient data of both study cohorts, the exact rate in the PU group could remain underreported, given the lack of laboratory results of the entire cohort. Another important factor is the significant higher rate of guidewire-induced perforation in the PTFE-CS group as compared to PU-covered group. Coverage of a side-branch with distal perforation by means of implantation of a CS in the main vessel, consequently, results in periprocedural MI.

In line, differences in periprocedural MI rates may be attributable to different procedural aspects, prolonged balloon inflation, septal- or side-branch occlusion, PE and tamponade, and therefore may not reflect device efficacy.

Finally, the predominant concern after implantation of early generation CS is the high incidence of acute stent failure. Previous studies reported stent thrombosis rates ranging from 5–30% within the first 6 months after early generation sandwich design PTFE-CS implantation in native coronary arteries or saphenous vein grafts. [16, 17, 19, 21, 22] Delayed endothelialization, high device thickness, delayed start, and early discontinuation of DAPT along with suboptimal device delivery and apposition have been attributed as explanation for the high incidence of thrombotic events after early generation PTFE-CS implantation. Recently we reported results of a retrospective study of new-generation single-layer PTFE-CS including 61 patients with no thrombotic event at 279 ± 197 days of follow-up [7]. On the other hand, the SOS PK Papyrus trial reported ST rates up to 10% after implantation of a new-generation single-layer PU-covered stent. These results have been attributed in part to the high proportion of emergency procedures, 50% of the population was admitted initially for MI, and the low rates of CS postdilatation [10]. Of note, a high proportion of patients underwent implantation of CS due to coronary aneurysm (15%) in this trial. Noteworthy is the low incidence of stent thrombosis in the current study, excluding patients with coronary aneurysms. Our data suggest an improved healing process after implantation of a new-generation PTFE- and PU-CS, irrespective of the cover material. Although, clinical presentation in the PU group was also MI in 50% of cases and significantly more frequent as compared to PTFE-CS group (20%), this difference did not result in a difference regarding antithrombotic efficacy. However, considering the delayed endothelialization after PTFE- or PU-CS implantation, prolongation of DAPT might be mandatory to prevent late thrombotic events. Patients in this study received different recommendation for prolonged duration of treatment after stenting and complete data relating to compliance or actual duration of DAPT are not available. An overall definite ST rate of 1.8% at 12 months, however, reflects an improved safety profile of both devices with regard to thrombotic events, notwithstanding also in part attributable to potent P2Y12 inhibition achieved with modern DAPT.

### Limitations

The main limitation of the present study is that it was a retrospective registry analysis. However, due to the low incidence of coronary artery perforation, the number of patients investigated is modest and it remains difficult to design a larger prospective randomized trial. Overall data on new-generation PTFE-based devices are limited and our study is the largest comparison of new-generation CS with different cover material currently available. Despite adjustment for potential differences between the two study cohorts, we are not able to rule out potential confounders concerning procedural characteristics. This is noteworthy, given the emergent character of the procedures during coronary perforation. Therefore, acute management might differ between individual patients, operators, centers, and countries in particular given the paucity of presentation and the heterogeneity of procedures which may not be displayed in the current analysis.

## Conclusion

In this study, a strategy of implantation of a new-generation single-layer PTFE- or PU-CS for the treatment of coronary artery perforation showed high success rates.

High but comparable revascularization rates mirror the demanding aftermath of coronary perforations. Both new-generation CS showed favorable and similar clinical safety, in particular, with regard to thrombotic events.

## Supplementary Information

Below is the link to the electronic supplementary material.

Supplementary file1 (DOCX 15 KB)

## Data Availability

Data are availabe after reasonable request.
