# Supplementary material for: Comparative safety and efficacy of new-generation single-layer polytetrafluorethylene- versus polyurethane-covered stents in patients with coronary artery perforation for the RECOVER (REsults after percutaneous interventions with COVERed stents) Investigators
Source: Cardiovasc Interv Ther. 2025 Feb 7;40(2):296–305. doi: 10.1007/s12928-025-01084-y (PMC11910408; doi:10.1007/s12928-025-01084-y)
Supplement: Supplementary file 1 — Supplementary file1 (DOCX 15 KB) [file 12928_2025_1084_MOESM1_ESM.docx]

**Supplemental Table 1: Recruiting centers**

**PTFE-covered stent (BeGraft-Coronary) implanting Centres**

- 1. Deutsches Herzzentrum München, Technische Universität München, Munich, Germany
  2. Department of Internal Medicine 2, University of Erlangen, Erlangen, Germany
  3. Universitätsherzzentrum Bad Krotzingen, Kardiologie 1, Bad Krotzingen, Germany
  4. 1.Medizinische Klinik, Klinikum rechts der Isar, Technische Universität München, Munich, Germany
  5. Herzzentrum der Segeberger Kliniken GmbH, Bad Segeberg, Germany

**PU-covered stent (Papyrus) implanting centres**

- 1. Herzklinik Ulm, Ulm, Germany
  2. Lund University, Skane University Hospital, Lund, Sweden
  3. Sunninghill Hospital, Johannesburg, South Africa
  4. University Hospital Ulm, Ulm, Germany
  5. Medical Campus Lake Constance, Friedrichshafen, Germany
  6. Institute of Cardiology, Warsaw, Poland
  7. Wroclaw Medical University, Wroclaw, Poland
  8. University Heart Center Hamburg, Hamburg, Germany
  9. University Heart Center Freiburg, Freiburg, Germany
  10. Hospital Fribourg, Fribourg, Switzerland
  11. Kliniken Sindelﬁngen, Sindelﬁngen, Germany
  12. Cardioangiologisches Centrum Bethanien, Franfurt, Germany
  13. CHUV (Centre Hospitalier Universitaire Vaudoise), Lausanne, Switzerland
  14. Groupe Hospitalier Mutualiste de Grenoble, Grenoble, France
  15. Clinique Pasteur, Toulouse, France
